# Crossing the digital frontier: are older adults ready for virtual reality workouts?

**DOI:** 10.3389/fpubh.2024.1324004

**Published:** 2024-02-08

**Authors:** André Ramalho, Pedro Duarte-Mendes, Rui Paulo, João Serrano, João Petrica

**Affiliations:** ^1^Sport, Health & Exercise Research Unit (SHERU), Polytechnic Institute of Castelo Branco, Castelo Branco, Portugal; ^2^Sport Physical Activity and Health Research & INnovation CenTer, SPRINT, Santarém, Portugal

**Keywords:** virtual reality, aging, physical activity, cognition, emotions

## Abstract

In the dynamic web of our digital age, virtual reality (VR) is crystallizing as a promising medium for promoting physical activity in older adults, overcoming age-old barriers. This perspective article explores the intricate synergy between VR and physical activity in older adults and highlights the many challenges of aging within a virtual environment. While VR heralds the potential for multisensory interaction and cognitive enhancement, a thorough assessment of its impact is paramount. The reach of VR goes beyond immediate applications and permeates the emotional and psychological realms of the human journey. Such an exploration requires a comprehensive perspective that goes beyond a purely technical assessment. The aim of this article is not to present clear-cut solutions, but to encourage reflection on the diverse impact of VR on our aging population. We argue for a future in which technology complements, rather than eclipses, the depth of human experience.

## 1 Why let the kids have all the fun? Older adults suiting up for VR!

In the realm of digital innovation, VR—a groundbreaking synthesis of immersive and interactive real-time experiences (1)—is increasingly serving as a bridge between generations. Having initially captivated a younger audience, VR is now poised to significantly impact the lives of our aging population (2) and provide a much-needed antidote to their sedentary lifestyles (3, 4). Thus, VR has evolved from a mere technological novelty to an important tool for improving the well-being of older adults (5, 6). However, the increasing acceptance of VR among this population raises a crucial question: Can VR be a universal panacea for their diverse needs, or are its benefits limited by practical factors such as affordability and ease of use? This question underlines the need for a nuanced assessment of the use of VR in geriatrics, whereby its innovative potential must be set against practical and economic considerations.

VR technology heralds a profound shift in human-computer interaction by immersing users in carefully crafted digital worlds that go beyond traditional real-world experiences. This evolution is due to head-mounted devices such as Oculus, HTC-Vive and Valve Index, which create a comprehensive, immersive 360-degree panorama and fully engage the user’s cognitive abilities. The essence of this interaction lies in its multidimensional nature, which is further enhanced by the inclusion of remote controls, haptic feedback and body gestures. These components blend together to transfer the user’s sensory and kinetic experiences into the VR environment. VR also plays a central role in the burgeoning field of mixed reality, where the boundaries between the virtual and real worlds are becoming increasingly blurred. Systems such as CAVE (Cave Automatic Virtual Environment) and CAREN (Computer Assisted Rehabilitation Environment) are an example of this fusion. They offer immersive 3D visualizations that simulate real-life scenarios in controlled environments (7).

In contrast, non-immersive technologies such as Microsoft Kinect and Nintendo Wii limit the user’s interaction to a conventional flat screen. As Šlosar et al. (7) argue, these modalities fall into the “PC category” because they limit the user’s experience to the physical confines of the screen and avoid the full sensory immersion characteristic of VR. The hallmark of VR is its ability to provide deep sensory immersion and create the illusion of a physical presence in an alternative environment. This feature clearly distinguishes VR from the more rudimentary, two-dimensional interactions of non-immersive technologies and forms a crucial frontier in the development of virtual interaction paradigms (7, 8).

VR-based home exercise programs are revolutionizing the field of physical activity, redefining traditional fitness paradigms and offering a range of new opportunities for older people (9). These programs, which include a range of commercially available games and applications, have been carefully designed to promote physical activity at home. They offer an arrow of virtual experiences, from bike rides to rhythmic dancing, all of which are possible with VR headsets such as Oculus Rift or HTC Vive. Participants are immersed in choreographed activities within these digital worlds. Immersive games such as Beat Saber, for example, ingeniously combine entertainment with physical exertion and allow older adults to participate in invigorating activities in an engaging way (10). These VR exercises go beyond traditional exercise routines by integrating interactive, game-like elements that increase user engagement and add a touch of playfulness to the fitness program. This innovative approach is particularly important for older people, for whom traditional forms of exercise can be challenging due to physical limitations.

A critical question arises when considering the digital recreations of real experiences through VR, such as virtual walks through Venice or simulated journeys through the Grand Canyon: Do these VR experiences really capture the multidimensional depth and complexity of real-life experiences? It is crucial to understand the emerging nature of VR, particularly in its application to enhance physical activity and motor skills. Paravlic et al. (11) emphasize the nascent stage of VR in enhancing physical activity and motor skills, noting that the current discourse on its effectiveness is mainly due to a lack of comprehensive research. They also emphasize that there is an urgent need for in-depth research on the wider effects of VR on emotional and psychological well-being. Furthermore, it is important to note that numerous studies include both immersive and non-immersive technologies, although this classification is often ambiguous (7).

Aldous Huxley’s observation that “technological progress has merely provided us with more efficient means of going backwards” (12) resonates in this context. It challenges us to consider whether innovations such as VR truly enrich our life experiences or merely create an illusion of progress. Our investigation must go beyond the superficial challenges of cost efficiency and usability. We need to go deeper and ensure that technological progress contributes positively to our fundamental human experiences, the core of our shared heritage. Ideally, technology should enhance our humanity, not diminish it.

In this perspective article, we explore the complex relationship between VR and the physical activity of older adults in the digital age. Rather than offering clear-cut solutions, we discuss the multiple challenges and perspectives on this pressing issue. As VR becomes more integrated into our lives, it is important to understand its nuanced impact on older adults and find a way forward. Our common goal? To create a digital world where every generation, especially the aging population, can utilize the full potential of VR. We are committed to promoting holistic well-being in a world that values the depth and richness of the human experience.

## 2 Ready, set, VR! The senior sprint into a healthier digital frontier

The digital age is changing the narrative of aging, particularly in the area of physical activity. In the past, the latter stages of life were perceived as a muted climax following the vibrant chapters of youth, but VR offers a new perspective that highlights the latent potential of the golden years. This technology goes beyond its original reputation and positions itself as a versatile tool that impacts older adults’ physical, cognitive, and emotional well-being (13). With VR, this population can pursue activities once considered unattainable—from climbing virtual mountains to practicing Tai Chi amid tranquil digital forests.

A burgeoning trend is the merging of interactive VR games with physical activity, known as “exergames.” These activities, ranging from simulated sports to dance routines, unfold in a digital world, creating a sensory-rich environment that seamlessly blends physical activity and recreation. By allowing embodied experiences, VR exergames sustain engagement, immerse older adults in virtual processes, and increase the likelihood that they will remain active longer (9, 14). The fusion of such virtual programs and exergames is proving to be an effective strategy that promotes engagement, ignites motivation, and improves health outcomes (15, 16). Although these approaches have the potential to promote healthier lifestyles among older adults, further research is needed to confirm their effectiveness for this population.

Traditional barriers—whether for health, logistical, or psychological reasons—have often limited senior mobility. In the context of our discussion, VR emerges as a tool that breaks down barriers and transforms everyday spaces into borderless activity areas. Academic research has confirmed the tangible benefits of VR-assisted physical activity. Tailored exercises promise both safety and a vibrant environment (17). These innovative approaches have revitalized many older adults by improving balance, mobility, and cardiovascular vitality (13). In addition, VR has shown that interactive training with visual feedback can outperform traditional physical training in improving functional mobility and balance in older adults. The multiple feedback and engaging nature of VR training could make it more effective and motivating, highlighting its distinct advantages in geriatric care and rehabilitation (18, 19).

The potential of VR goes beyond physical well-being and offers a mix of cognitive challenges and physical activity. With VR, older adults can explore historical sites and unfamiliar terrain, combining memory, spatial awareness and movement into a neurocognitive exercise. Emerging research substantiates that the multisensory engagement offered by modern virtual interfaces can significantly improve cognitive abilities (20, 21). This form of interaction has been shown to outperform the effectiveness of non-immersive technologies such as Nintendo Wii or Microsoft Kinect (7, 10), suggesting superior potential for cognitive stimulation and development.

In addition, the role of VR in the prevention of neurodegenerative diseases such as Alzheimer’s is gaining scientific interest, as it offers benefits for both cognitively healthy people and those with cognitive decline (22, 23). The field of human-computer interaction, particularly in the area of rehabilitation, has undergone a profound transformation due to advances in sensor technology (24). Current scientific research highlights the effectiveness of these sophisticated sensors in customizing user experiences through careful monitoring of physiological responses. This development highlights the central role that digitally based interventions play in providing a controlled environment in which the benefits of physiological human-computer interaction can be explored. Such innovative methods are crucial for the development of rehabilitation strategies that are both personalized and adaptive (7, 9, 25). Older adults have also been shown to consistently participate in VR, as evidenced by minimal dropout rates, further underscoring the appeal of this modality (10). Given the integrated benefits of physical activity and cognitive enrichment offered by VR, the question arises: could VR become a central tool for maintaining cognitive abilities in old age?

The potential of VR is not limited to physical and cognitive areas. Many older adults, no matter where they live, struggle with the challenges of sedentary lives and limited environments. VR offers a solution by making accessible places previously considered distant or inaccessible (13). Virtual journeys, whether through quiet forests or along tranquil riverbanks, offer more than sensory pleasures. They encourage movement, stimulate curiosity, and challenge the pervasive sedentary tendencies that plague the aging demographics (26). Combining VR with the essence of nature paves the way for holistic enrichment. This fusion of cutting-edge technology and the therapeutic essence of nature opens new avenues for combating isolation and creating revolutionary therapeutic paradigms (27, 28).

In essence, VR is not just a testament to technological progress. It holds the potential to rewrite the history of aging by presenting it not as a muted conclusion to the vivid sagas of youth, but as a chapter full of dormant possibilities. By harnessing the physical, cognitive, and emotional resonance of VR, we are on the cusp of a significant challenge: to redefine our perception of aging and expand our understanding of human potential across the lifespan. With this in mind, an important question arises: Are we on the cusp of a paradigm shift in which technology not only shapes our twilight years, but also fundamentally changes our perception of aging?

## 3 Putting the “real” in virtual: inclusion, accessibility, and maybe some dance parties!

In the ever-evolving mosaic of technology, we are tasked with recalibrating our conceptual framework, especially concerning the accessibility and autonomy of the aging population. This population is in a poignant interplay between youthful aspirations and the constraints of the present (26). Traditional institutions such as nursing homes provide structured care, but they can inadvertently convey limitations and overshadow memories of more vibrant times. In this context, VR proves to be a bridge that provides both temporal and spatial emancipation. It makes once-traveled mountain paths and fond memories of the sea accessible, even from the confines of a nursing home (27).

While we marvel at these possibilities, we must go deeper: Can VR really be the great equalizer, removing age-related limitations and granting the intellect its freedom? Could VR, reflecting Simone de Beauvoir’s insatiable longing for the full spectrum of life, offer older adults a way to bridge the gap between tangible reality and their latent desires? As we move into this realm, our mission becomes clear: technology should not only facilitate physical revitalization, but also act as a conduit that underscores the essence of the human experience and ensures that the soul remains alive over time. Amidst the tangled web of technological advancement, VR emerges brightly, connecting our reality with the limitless digital worlds. Echoing Plato’s allegory of the cave, VR opens up new vistas previously limited by physical constraints. The dawn of this era, however, also raises questions of authenticity: Are these virtual experiences just a shadow or do they have comparable value to their real-life counterparts?

Undoubtedly, VR offers therapeutic opportunities that allow the aging population to engage in activities they previously avoided. The innovative integration of virtual natural environments clearly increases older adults’ motivation to exercise, as evidenced by their increased participation in virtual cycling, for example (19). This technological alchemy captures the rejuvenating essence of nature and makes it accessible even to people with pronounced physical limitations (28). It entices us to imagine a world where the boundaries between reality and the digital are blurred and our understanding of limits is redefined. Moreover, VR invites us to a profound philosophical reflection on the nature of reality, the inexorable passage of time, and our existential limits. We face a double challenge: to maximize VR ‘s potential for connection and enrichment while preserving the sanctity of genuine human bonds.

The ongoing development of VR technology heralds a crucial threshold in enhancing the sensory experiences of older people, particularly through the integration of new technologies such as haptic feedback and olfactory sensations. These developments aim to create a VR environment that goes beyond traditional visual and auditory limitations and strives for a more holistic and interactive virtual world. This shift towards enhanced multisensory engagement significantly increases the realism and appeal of VR for older adults, enabling a deeply personal and immersive virtual experience. Such technological improvements play a crucial role in the success of VR interventions. They can enhance the positive effects in areas such as motor and cognitive rehabilitation while mitigating negative effects such as cybersickness (7). Therefore, the fusion of these sensory enhancements in VR is crucial, not only to bridge the gap between the digital and natural worlds, but also to realize the full therapeutic potential of VR for the geriatric population. This emerging synthesis heralds a new era of exploration and innovation, making it an important focus area for ongoing research.

In a world where social isolation among older adults is on the rise, VR is a beacon that revives the community bonds of the past. It offers more than digital panoramas; it creates spaces that encourage interaction, kinetic activities, and stimulating discussions (13, 28). Applications such as Alcove VR, Meta Quest 2 or EngageVR epitomize this, offering virtual environments where older adults can gather, participate in diverse activities, and share experiences, thereby facilitating meaningful connections. These digital spaces go beyond just fitness platforms and are designed to appeal to the aging demographics, especially those who struggle with isolation and physical limitations (16). For VR to be truly integrated into their lives, it should be responsive to and accommodate their diverse experiences. And most importantly, the accessibility of VR—both financially and in terms of usability—is critical to true inclusion (17). In this way, VR not only counteracts social isolation, but also provides a sense of community and confirms its role as a tool for enriching the lives of our older population.

In summary, VR is an example of the symbiosis of social development and technological innovation, especially in its integration with older adult care. It offers profound humanistic potential: empowerment, integration and introspection. For older adults, it is more than just a digital stay; it awakens their passion for precious moments. In this digital age, our mission is clear: to seamlessly connect tangible moments with subtle worlds and cultivate authentic human relationships amidst the digital landscape. Over time, VR has the potential to serve as a poignant hub connecting the memories of days gone by, the realities of the present, and the promise of an uncertain future.

## 4 Silver-haired hiccups in the virtual landscape!

Incorporating VR into elder care brings a wealth of opportunities and challenges. Although digital technology holds promise for promoting physical activity among older adults, caution is warranted. Over-enthusiasm in introducing such technologies is reminiscent of the Icarus tale. Problems such as dizziness or fatigue with prolonged use of VR represent only a fraction of the potential problems (29, 30). As VR becomes more widespread, maintaining quality becomes a critical factor. These digital worlds should not only entertain but must also be designed with therapeutic efficacy and user safety in mind.

Beneath the surface, the psychological implications of VR are profound. The ability of VR to reawaken dormant memories and emotions requires a sensitive and thoughtful approach. Older adults’ varying familiarity with digital tools further complicates the issue and heightens concerns about potential emotional disconnection or distorted perceptions of reality (30). It is important to find a balance that ensures this population maintains tangible human relationships while navigating virtual worlds. The integration of VR with modern technological advances and wearable health devices also raises ethical dilemmas. It is important to limit content, control screen time, and ensure age-appropriate content. Preliminary studies suggest the potential of VR (13), but there is an urgent need for detailed, long-term research to fully assess its impact on the aging population.

Given our innate connectedness to the natural world—underscored by the biophilia hypothesis (31)—one wonders if a digital facsimile such as VR can truly reproduce the rhythm of nature or the warmth of human interaction. In this age of technological marvels, we are in danger of becoming detached from our elemental roots. Older adults’ varied responses to VR reinforce this problem. VR holds the most promise when it matches experiences in the real world, but it should complement and reinforce, not replace, our innate connections to the world and each other through physical activity.

Equity and accessibility are also of paramount importance. What about the accessibility of VR to all economic classes? And how does it compare to traditional physical activities tailored to older adults? Research offers a cautiously optimistic outlook for VR in elder care. For VR to gain acceptance, it must be scientifically based and bridge the gap between theory and practice (3). In addition, cultural nuances play a critical role. Some individuals may perceive VR as a disruption of their traditional values, while others may see it as a bridge between the past and the present. The anticipated merging of artificial intelligence with VR will enable the creation of customized virtual experiences, each tailored to an individual’s unique story. Most importantly, VR could serve to bridge the generation gap by enabling shared experiences between older and younger generations.

When digital innovation and time-honored prudence collide, the metaphor of a budding sapling comes to mind. As stewards of our collective future, can we nurture the roots of VR so that they transcend mere technological growth and flourish in harmony with our human nature? Could VR serve as fertile soil that enables older adults to grow beyond mere existence and weave the tangible and the digital into a timeless fabric of connectedness? Our point is clear: VR holds significant potential—especially when tailored to older adults — and should complement, not overshadow, interpersonal relationships. This ethos aligns with John Naisbitt’s assertion that the innovations of our time will depend more on profound human insights than on pure technological capabilities. In our pursuit of digital progress, the essence of our humanity must remain sacrosanct.

At this point, the question inevitably arises: Are older adults really ready to integrate VR-enhanced physical paradigms into their lives? From our deliberations, it appears that the promise of VR—with its potential to enrich older adults’ physical, cognitive, and emotional domains—is subject to complex considerations of acceptability. The confluence of technological advances and elder care is less about the functionalities of VR and more about adapting to seniors’ sensibilities. Such an endeavor requires a keen awareness of the delicate interplay between the use of cutting-edge solutions and the preservation of genuine human relationships. When thinking about the integration of VR into elder care, a holistic vision emerges—one that not only extols the wonders of technology, but also honors the enduring intricacies of human relationships and emotions. Ultimately, the acceptance of VR by our older generation and its effectiveness will depend on the ability to combine technological innovation with a deep respect for the human touch in elder care.

## Data availability statement

The original contributions presented in the study are included in the article/supplementary material, further inquiries can be directed to the corresponding author.

## Author contributions

AR: Writing – original draft, Writing – review & editing. PD-M: Formal analysis, Writing – review & editing. RP: Formal analysis, Writing – review & editing. JS: Formal analysis, Writing – review & editing. JP: Supervision, Writing – review & editing.
